# Gamified Augmented Sensorimotor Feedback During Balance Training Improves Postural Control and Nocturnal RMSSD

**DOI:** 10.3390/jfmk11040414

**Published:** 2026-10-10

**Authors:** Vishruth Shatagopam, Jake Pirnazar, Phillip Goldman, Trent Yamamoto, Joshua A. Kidwell, Brett A. Dolezal

**Affiliations:** 1 Airway and UC Fit Digital Health-Exercise Physiology Laboratory, David Geffen School of Medicine, University of California Los Angeles, Los Angeles, CA 90024, USA; jakep007@g.ucla.edu (J.P.); phillip.goldman@bcm.edu (P.G.); tyama@bu.edu (T.Y.); joshuakidwell@creighton.edu (J.A.K.); 2 Baylor College of Medicine, Houston, TX 77030, USA; 3 Chobanian and Avedisian School of Medicine, Boston University, Boston, MA 02118, USA; 4 School of Medicine, Creighton University, Omaha, NE 68178, USA; 5 The Fitness Prof, Los Angeles, CA 90001, USA

**Keywords:** balance training, augmented feedback, heart rate variability, RMSSD, postural control, gamification

## Abstract

**Background and Objectives**: Augmented sensorimotor feedback may enhance motor learning during balance training, but whether these adaptations extend to cardiac autonomic regulation remains unclear. This study compared wobble-board training with gamified augmented feedback versus conventional wobble-board training on dynamic balance and nocturnal heart rate variability. **Methods**: Thirty adults were assigned to training with gamified-augmented feedback (GBT), conventional training (CBT), or control (CON). GBT and CBT completed three ~20-min sessions weekly for six weeks. Dynamic balance, measured via the modified Star Excursion Balance Test (mSEBT), and nocturnal Root Mean Square of Successive Differences (RMSSD) were assessed. **Results**: Group × time interactions occurred for RMSSD and mSEBT (both *p* < 0.001). RMSSD increased 6.93 ± 1.86 ms in GBT, versus 1.01 ± 1.38 and 0.80 ± 1.61 ms in CBT and CON, respectively. GBT exceeded CBT (mean difference, 5.92 ms; 95% CI, 4.37–7.46; Hedges’ g = 3.46) and CON (6.12 ms; 95% CI, 4.49–7.76; g = 3.38; both adjusted *p* < 0.001); CBT did not differ from CON (*p* = 0.761). mSEBT increased 7.54 ± 1.62 points in GBT, 3.65 ± 1.50 in CBT, and 0.47 ± 1.46 in CON. GBT exceeded CBT (mean difference, 3.89 points; 95% CI, 2.42–5.36; g = 2.39) and CON (7.07; 95% CI, 5.63–8.52; g = 4.40), while CBT exceeded CON (3.18; 95% CI, 1.79–4.57; g = 2.06; all adjusted *p* < 0.001). **Conclusions**: Gamified augmented feedback and its associated features were associated with greater improvements in dynamic balance and RMSSD than conventional training. Mechanisms underlying RMSSD response require further investigation.

## 1. Introduction

Postural control is fundamental to human movement, contributing to functional independence, injury prevention, and physical performance. This complex sensorimotor process integrates visual, vestibular, and proprioceptive information to maintain or restore the body’s center of mass during activity [1]. Because balance depends on coordinated neural, sensory, and musculoskeletal function, it represents an important indicator of integrated sensorimotor function. As falls remain a leading cause of injury-related morbidity and mortality among adults aged 65 years and older [2,3], it is of paramount importance to preserve balance as our population ages. Exercise-based interventions have become a cornerstone of balance rehabilitation and are among the most effective strategies for reducing falls, decreasing fall rates by approximately 23% [2].

Although balance training is frequently associated with rehabilitation and aging populations, postural control is equally important for healthy, recreationally active adults. Efficient balance contributes to movement quality, force transmission through the kinetic chain, agility, and the ability to respond rapidly to both anticipated and unexpected perturbations during physical activity [4,5,6,7,8]. Deficits in postural control have also been associated with an increased risk of musculoskeletal injury, with poor performance on balance assessments predicting a greater likelihood of noncontact lower-extremity injury [9]. Consequently, interventions that enhance postural control may have broad applications for both injury prevention and physical performance.

Conventional balance training improves postural stability through enhancing proprioceptive function [10], neuromuscular coordination [11], and sensorimotor integration [12]. Wobble-board training is employed to expose individuals to unstable conditions, promoting repeated sensorimotor adaptation and improving both static and dynamic balance [13,14]. Despite these benefits, conventional balance training is often task-specific and may provide limited external feedback, potentially constraining motor learning, participant engagement, and transfer to untrained activities [15,16]. These limitations have prompted increasing interest in interventions designed to augment feedback, motor learning, and participant engagement during balance training.

Motor-learning theory proposes that augmented feedback—information provided from an external source in addition to intrinsic sensory feedback—facilitates movement correction, skill acquisition, and long-term retention [17]. Advances in wearable sensors and interactive technologies now allow real-time augmented feedback through audiovisual cues, objective performance metrics, and progressively challenging tasks. Gamified balance training combines these features within an interactive environment that requires continuous adjustments of the center of mass in response to external visual stimuli. By integrating physical and cognitive demands, this approach may enhance sensorimotor learning, promote cortical and subcortical adaptations, and improve motivation and adherence to training [18].

Beyond improvements in postural performance, cognitively demanding exercise may also influence cardiac autonomic regulation. Heart rate variability (HRV), particularly the root mean square of successive differences between normal R–R intervals (RMSSD), is a widely used noninvasive marker of cardiac parasympathetic modulation [19]. Higher resting RMSSD generally reflects greater vagally mediated cardiac autonomic modulation and has been used extensively to characterize autonomic input and exercise adaptation [20,21]. Although the effects of aerobic and resistance exercise on HRV have been extensively investigated, comparatively little is known regarding whether balance training, particularly when enhanced by augmented sensorimotor feedback, can produce favorable cardiac autonomic adaptations.

To our knowledge, no previous study has directly compared gamified and non-gamified wobble-board training while simultaneously evaluating postural performance and cardiac autonomic regulation in recreationally active adults. Examining these outcomes together may provide important insight into whether augmented sensorimotor feedback produces broader physiological adaptations than conventional balance training alone. Thus, the purpose of the present randomized exercise intervention study was to compare the effects of six weeks of thrice-weekly gamified wobble-board training with conventional wobble-board training on dynamic postural control and vagally mediated cardiac modulation, assessed using nocturnal RMSSD. We hypothesized that participants receiving real-time gamified augmented feedback would demonstrate greater improvements in dynamic balance together with more favorable vagally mediated cardiac modulation than participants performing conventional wobble-board training without augmented feedback.

## 2. Materials and Methods

### 2.1. Study Design

**Figure 1:**
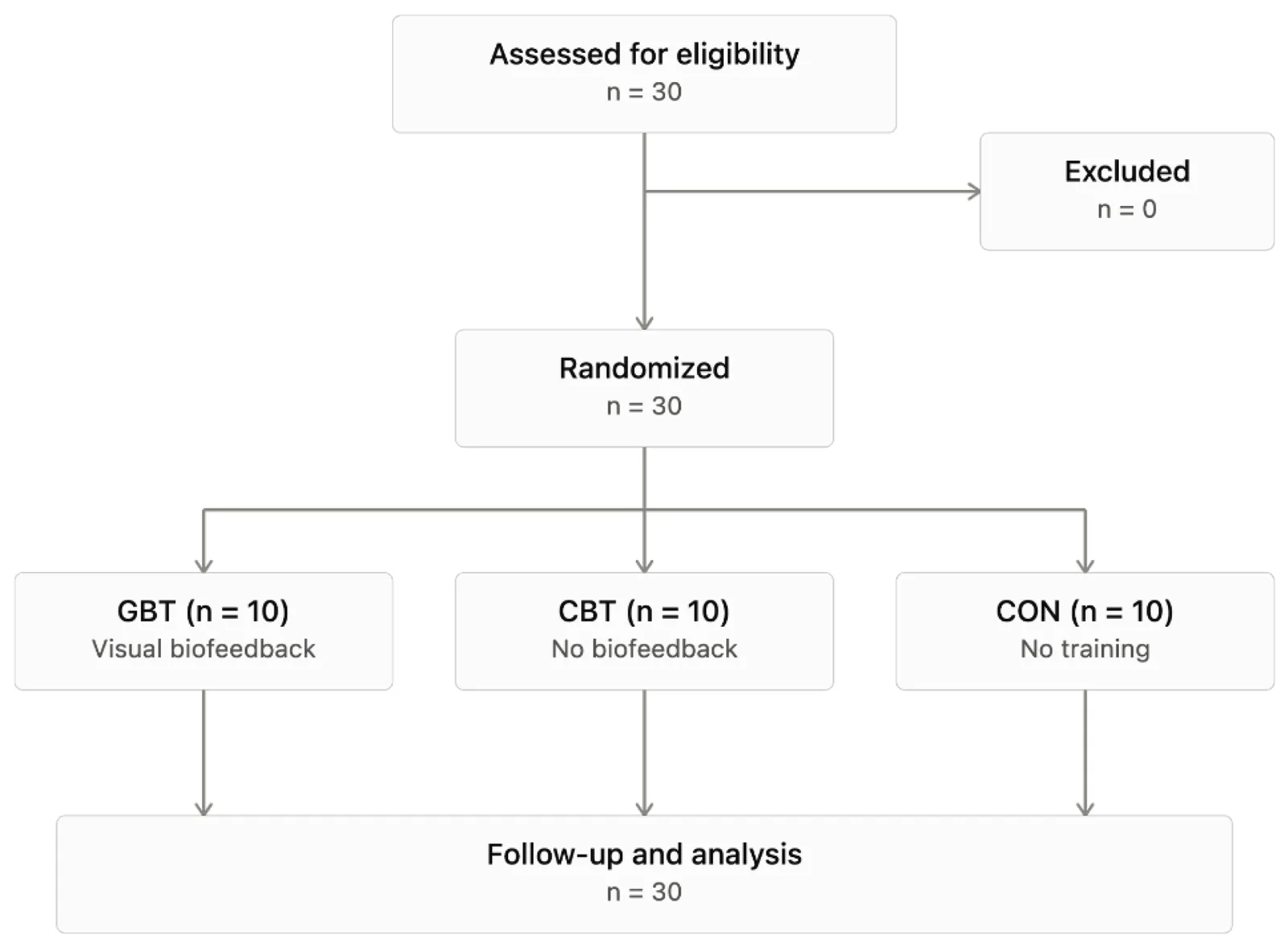
CONSORT participant-flow diagram. Claude [claude-sonnet-4-6 model, Anthropic] was used to assist with preparation of the diagram; the authors reviewed and verified the final content.

This six-week randomized exercise intervention study included 30 recreationally active adults (12 females) who were randomly assigned in equal numbers (*n* = 10 per group) to one of three experimental conditions (Figure 1): (1) gamified balance training with augmented sensorimotor feedback (GBT), (2) conventional balance training performed on the same balance board without interactive feedback (CBT), or (3) a non-training control group (CON). Group allocation was generated by an investigator independent of participant recruitment using an online random-number generator. The independent investigator retained the randomization list, and group assignments were concealed from personnel responsible for participant recruitment and baseline assessment until enrollment and baseline testing had been completed. Participants assigned to GBT and CBT completed three supervised training sessions per week for six consecutive weeks (18 sessions total), with each session lasting approximately 20 min. Outcome assessors remained blinded to group allocation throughout data collection.

### 2.2. Participants

Thirty apparently healthy, recreationally active adults volunteered to participate in the study. Recreational activity was defined as participation in structured exercise at least once per week during the preceding year. Participants were excluded if they had participated in formal balance training within the previous six months or had any cardiovascular, neurological, musculoskeletal, metabolic, vestibular, or pulmonary disorder that could impair balance performance, limit participation, or increase the risk associated with study participation.

Participants enrolled in the present randomized exercise intervention study provided written informed consent under a single Institutional Review Board (sIRB 11-003190: BRANY, Melville, NY, USA). All study procedures were performed at an independent fitness facility in the Greater Los Angeles metropolitan area in accordance with the ethical principles outlined in the Declaration of Helsinki. Participants were informed that they could withdraw from the study at any time without penalty.

Ethics approval was obtained on 5 June 2026. Recruitment and enrollment began concurrently on 8 June 2026, when participants received their Oura Rings and initiated the seven-night baseline HRV acquisition period. Following the completion of baseline monitoring, the six-week intervention began on 15 June 2026. Post-intervention assessment included the subsequent seven-night HRV acquisition period, and all final assessments were completed by 7 August 2026. The study was not prospectively registered in a public trial registry; at study inception, it was classified by the investigators as nonclinical exercise-physiology research in healthy adults.

An a priori sample-size calculation was performed using G*Power version 3.1. The calculation was based on a between-group comparison using an effect-size estimate derived from an independent exploratory pilot study of 10 collegiate-aged males who completed a similar balance-training intervention. The pilot-derived effect size was Cohen’s d = 1.30. Using a *t*-test (two-tailed, α = 0.05, allocation ratio 1:1, and 80% statistical power), the analysis indicated that a minimum of 10 participants per group was required. The pilot investigation was conducted independently from the present randomized exercise intervention study with Institutional Review Board approval (IRB No. 11-003190).

### 2.3. Balance Training Intervention

**Figure 2:**
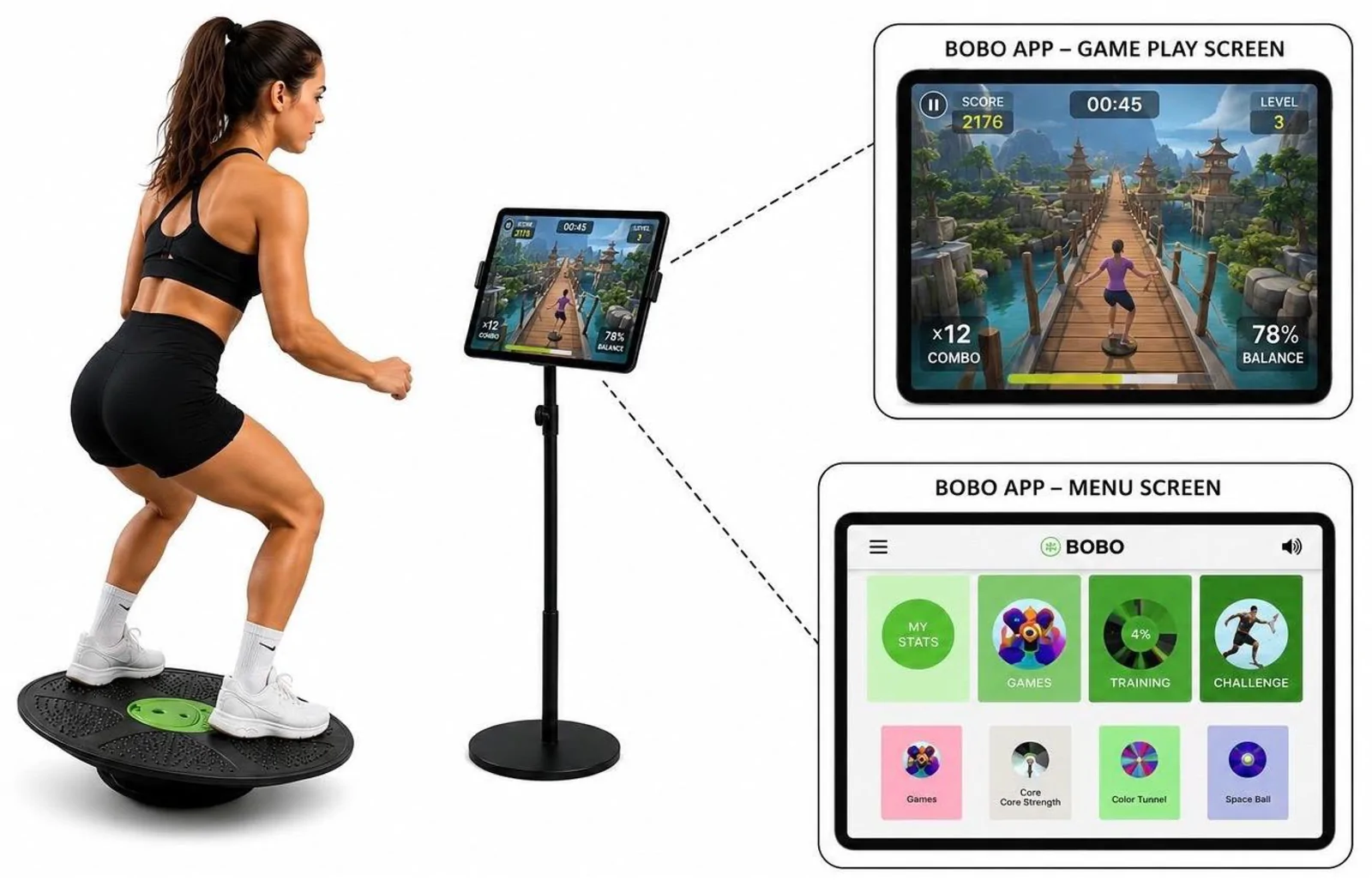
Illustrative representation of the BOBO Balance Board and application used during the study. The image was prepared with AI assistance and reviewed by the authors against the study equipment and software configuration for accuracy.

Participants assigned to the two intervention groups completed identical six-week balance-training programs consisting of three supervised sessions per week (18 sessions total). Each session lasted approximately 20 min and was performed on the same BOBO Balance Board (BOBO Balance, Tel Aviv, Israel). The BOBO Balance Board consists of a rigid circular platform supported by a hemispherical base, creating a multidirectional unstable support surface that permits controlled movement in the anterior–posterior, medial–lateral, and diagonal planes. Embedded inertial motion sensors continuously quantified board orientation and angular displacement associated with multidirectional postural movements. Both intervention groups completed identical exercise progressions using the same board, supervision, session duration, and training frequency. Representative examples of the BOBO Balance Board and the interactive software interface are presented in Figure 2. ChatGPT [GPT Image 2 model, OpenAI] was used for the purposes of generating Figure 2.

#### 2.3.1. Gamified Balance Training (GBT)

Participants assigned to the gamified balance-training group performed all exercises using the BOBO interactive balance-training system, which integrated the multidirectional balance board with the BOBO Home software application. Throughout every training session, the embedded inertial sensors continuously monitored board orientation and multidirectional movement while wirelessly transmitting these data to the application in real time.

The BOBO Home application translated participants’ postural movements into immediate visual biofeedback displayed on a tablet positioned directly in front of the participant. Rather than simply maintaining balance on the board, participants interacted with a series of interactive games and task-oriented exercises by shifting their center of mass to manipulate on-screen objects toward predetermined targets. Successful task completion required continuous integration of visual, vestibular, and proprioceptive information while rapidly modifying trunk position and lower-extremity loading to achieve progressively changing task objectives.

Throughout each session, the software provided immediate visual feedback, objective performance scores, movement-accuracy metrics, reaction-time feedback, postural-stability metrics, and task-completion scores. Exercise difficulty progressively increased throughout the intervention by requiring greater movement precision, faster reaction times, narrower stability limits, and increasingly complex postural-control strategies. The interactive environment required participants to identify movement errors and continuously modify their postural responses in accordance with the visual task demands.

#### 2.3.2. Conventional Balance Training (CBT)

Participants assigned to the conventional balance-training group completed the same six-week balance-training program using the same BOBO Balance Board, exercise progression, session duration, supervision, and training frequency as the gamified group. However, the BOBO Home application’s interactive features and all real-time visual biofeedback were disabled throughout every training session. Participants performed matched balance tasks on the identical unstable surface but received no external visual feedback, objective performance scores, movement-accuracy metrics, reaction-time feedback, targets, or game-based interaction. Instead, movement correction relied on intrinsic sensory information and naturally available environmental cues. Thus, the physical balance-training stimulus was matched as closely as practicable, while the bundled interactive gamified-feedback features were unique to GBT.

#### 2.3.3. Control Group (CON)

Participants assigned to the control group did not participate in any balance-training intervention during the six-week study period. They were instructed to maintain their usual physical activity and exercise habits while refraining from initiating any structured balance-training program or using instability-training devices. Compliance was verified through weekly communication with study personnel. Control participants completed all baseline and post-intervention assessments using the same testing procedures and schedule as the intervention groups.

#### 2.3.4. Standardized Balance Training Protocol

Participants assigned to the GBT and CBT groups completed identical exercise protocols that differed only in the presence or absence of gamified augmented sensorimotor feedback. Each supervised training session lasted approximately 20 min and consisted of a standardized progression of balance exercises designed to challenge postural stability under progressively greater levels of difficulty. Exercises included bilateral and unilateral stance tasks performed in the anterior–posterior, medial–lateral, and multidirectional planes while maintaining an upright posture with the arms comfortably positioned at the sides.

Exercise difficulty progressively increased throughout the six-week intervention by systematically reducing the base of support, increasing movement precision, introducing multidirectional weight-shifting tasks, and incorporating progressively more demanding postural-control activities. Progression was individualized, but GBT and CBT were matched as closely as practicable for training frequency, session duration, supervision, board instability, movement direction, time-on-task, and weekly progression. Participants advanced after successful completion of the assigned task while maintaining appropriate postural control. CBT activities were selected to reproduce the principal physical and postural demands of the corresponding GBT games. For example, a four-minute GBT spaceship task emphasizing medio-lateral control and contralateral weight shifting was matched by four minutes of CBT movements emphasizing the same stabilization demands. The standardized six-week progression is summarized in Table 1.

Table 1 standardized progression of the six-week balance-training program completed by the GBT and CBT groups. Both groups were matched as closely as practicable for exercise progression, supervision, and training volume. The interactive gamified augmented-feedback environment and its associated features were unique to GBT.

### 2.4. Outcome Measures

Primary outcome measures included nocturnal HRV quantified using RMSSD and dynamic postural balance assessed using the mSEBT. All assessments were performed before and after the six-week intervention under standardized testing conditions by investigators blinded to group allocation.

#### 2.4.1. Heart Rate Variability

Cardiac vagal modulation was assessed using nocturnal heart rate variability (HRV) recorded with the Oura Ring Generation 4 (Oura Health Oy, Oulu, Finland). Participants wore the device continuously throughout the study and were instructed to wear the ring during all overnight sleep periods. The Oura Ring utilizes infrared photoplethysmography (PPG) to measure pulse-wave signals during sleep. The Generation 4 ring has demonstrated excellent agreement with electrocardiographic reference measurements for nocturnal HRV [22]. The primary HRV outcome was the root mean square of successive differences between normal R-R intervals (RMSSD), a widely used time-domain index of vagally mediated cardiac modulation. The device derives nightly RMSSD from multiple signal-quality screened and artifact-filtered measurements collected throughout sleep, reducing the influence of daytime posture and activity on the measurement period.

Baseline nocturnal HRV was calculated as the mean RMSSD obtained across seven consecutive nights preceding the intervention. Nocturnal RMSSD continued to be recorded throughout the six-week study period, and post-intervention HRV was calculated as the mean RMSSD across seven consecutive nights following the completion of the intervention. All 30 participants contributed seven valid nights of nocturnal RMSSD data during both the baseline and post-intervention assessment periods (7/7 nights at each time point). Baseline and post-intervention RMSSD values therefore represented complete seven-night means for all participants. The primary HRV outcome was the change in seven-night mean nocturnal RMSSD from baseline to post-intervention. No missing-night or outcome-value imputation was required. Participants synchronized their Oura Ring data with the Oura mobile application regularly to facilitate data capture. Day-to-day behaviors such as caffeine or alcohol intake, nonstudy exercise, transient illness, and sleep behavior were not prospectively standardized or statistically adjusted.

#### 2.4.2. Dynamic Balance Assessment

Dynamic balance was assessed using the modified Star Excursion Balance Test (mSEBT), a valid and reliable measure of dynamic postural control and lower-extremity neuromuscular function [23]. The mSEBT was selected over the traditional eight-direction Star Excursion Balance Test because it provides a more time-efficient assessment while maintaining reliability and validity through evaluation of the anterior, posteromedial, and posterolateral reach directions [24]. To determine which leg was dominant, participants were asked which leg they would kick a ball with. During testing, participants stood barefoot on the dominant stance limb with the great toe positioned at the center of the testing grid and the line bisecting the second and third toes aligned with the anterior reach line, consistent with previously published guidelines. While maintaining their hands on their hips, participants reached maximally with the contralateral limb in the anterior, posteromedial, and posterolateral directions before returning to the starting position without compromising balance. Limb length was measured from the anterior superior iliac spine to the inferior border of the lateral malleolus, with the participant in a relaxed supine position.

Participants completed four familiarization trials followed by three recorded trials in each reach direction for the dominant limb. A trial was repeated if the participant moved the stance foot, transferred weight onto the reaching limb, removed their hands from their hips, or was unable to maintain postural control throughout the movement. Reach distance was measured in centimeters from the center of the grid to the point of maximal reach by the great toe of the reaching limb. To account for differences in limb length, reach distances were normalized to stance-limb length and expressed as a percentage. A composite reach score was subsequently calculated as the average of the normalized anterior, posteromedial, and posterolateral reach distances. The mean of the three recorded trials for each direction was used for statistical analysis [25]. Higher normalized reach distances and composite scores reflected superior dynamic balance performance.

### 2.5. Statistical Analysis

Data were examined for completeness, implausible values, and distributional assumptions before analysis. Continuous variables are reported as mean ± standard deviation (SD). Baseline demographic and outcome characteristics were compared among the gamified balance-training (GBT), conventional balance-training (CBT), and non-exercising control (CON) groups using one-way analysis of variance. The primary outcomes were the seven-night mean nocturnal root mean square of successive differences (RMSSD) and the modified Star Excursion Balance Test (mSEBT) composite score measured before and after the six-week intervention. Each outcome was analyzed using a linear mixed-effects model containing fixed effects for group, time, and the group × time interaction, with a participant-specific random intercept to account for within-subject correlation across the repeated pre/post observations. The group × time interaction served as the primary omnibus test of whether pre-to-post change differed among groups. Model residuals were inspected for normality, and homogeneity of change-score variance was evaluated using Levene’s test. When a significant group × time interaction was identified, prespecified between-group differences in individual change scores were examined using Welch independent-samples tests. Welch tests were selected because they do not require equal between-group variances and provide a robust follow-up comparison when variance equality may be uncertain in small groups. Holm adjustment controlled the family-wise error rate across the three pairwise comparisons. Standardized within-group effects were expressed as Cohen’s dz, whereas between-group differences in change were expressed as Hedges’ g with 95% confidence intervals (CIs). All 30 randomized participants were included in the primary analyses; no imputation was required for the primary pre/post outcomes. Statistical significance was set at *p* < 0.05. Analyses were performed using SAS 9.4M9.

## 3. Results

All 30 randomized participants (12 females) completed the study. No adverse events (e.g., injury) or protocol deviations occurred during training or testing. For the overall cohort, age was 20.5 ± 1.3 years, height was 170.9 ± 8.1 cm, body mass was 74.3 ± 7.4 kg, and BMI was 25.6 ± 3.3 kg·m^−2^. Baseline demographic and outcome characteristics for each randomized group are presented in Table 2. No significant baseline differences were observed among groups (all *p* > 0.05). Training adherence was 97.8% in both intervention groups.

A significant group × time interaction was observed for nocturnal RMSSD (Wald χ^2^(2) = 101.53, *p* < 0.001), indicating that pre-to-post changes differed among groups. RMSSD increased by 6.93 ± 1.86 ms in GBT, compared with 1.01 ± 1.38 ms in CBT and 0.80 ± 1.61 ms in CON. Holm-adjusted pairwise comparisons demonstrated greater improvement in GBT than CBT (mean difference, 5.92 ms; 95% CI, 4.37 to 7.46; adjusted *p* < 0.001; Hedges’ g = 3.46) and CON (mean difference, 6.12 ms; 95% CI, 4.49 to 7.76; adjusted *p* < 0.001; Hedges’ g = 3.38). Changes did not differ between CBT and CON (mean difference, 0.21 ms; 95% CI, −1.20 to 1.62; adjusted *p* = 0.761; Hedges’ g = 0.13).

**Figure 3:**
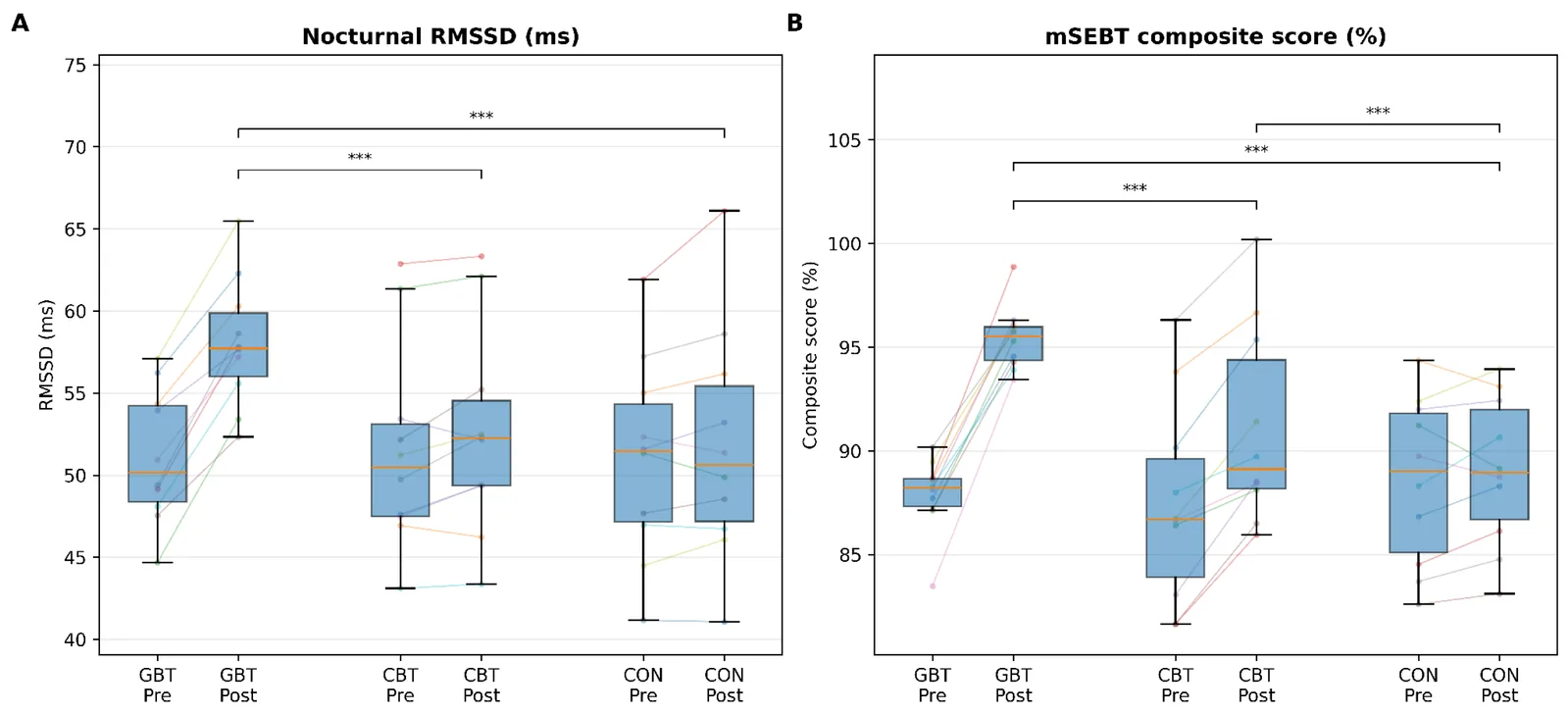
Longitudinal changes in nocturnal RMSSD and dynamic balance. Box-and-whisker plots depict pre- and post-intervention distributions for nocturnal RMSSD (**A**) and mSEBT composite score (**B**) in the gamified balance-training (GBT), conventional balance-training (CBT), and control (CON) groups. Connecting lines represent individual participant changes from baseline to post-intervention. Brackets denote significant Holm-adjusted between-group differences in change scores (*** *p* < 0.001).

A significant group × time interaction was also observed for the mSEBT composite score (Wald χ^2^(2) = 119.62, *p* < 0.001). Composite scores increased by 7.54 ± 1.62 percentage points in GBT, 3.65 ± 1.50 percentage points in CBT, and 0.47 ± 1.46 percentage points in CON. Holm-adjusted pairwise comparisons demonstrated greater improvement in GBT than CBT (mean difference, 3.89 percentage points; 95% CI, 2.42 to 5.36; adjusted *p* < 0.001; Hedges’ g = 2.39) and CON (mean difference, 7.07 percentage points; 95% CI, 5.63 to 8.52; adjusted *p* < 0.001; Hedges’ g = 4.40). CBT also improved more than CON (mean difference, 3.18 percentage points; 95% CI, 1.79 to 4.57; adjusted *p* < 0.001; Hedges’ g = 2.06). These changes are demonstrated in Table 3 and Figure 3.

## 4. Discussion

The present randomized exercise intervention study demonstrated that six weeks of gamified balance training incorporating an interactive augmented-feedback environment produced greater improvements in dynamic postural control and nocturnal RMSSD than conventional balance training and a non-training control condition. Conventional balance training also improved mSEBT performance relative to control; however, the magnitude of improvement was substantially greater when the matched physical balance-training stimulus was paired with the interactive gamified augmented-feedback environment. In contrast, the change in nocturnal RMSSD was greater in GBT than in both CBT and CON, whereas CBT did not differ from control. Because the GBT condition bundled real-time visual feedback, visual targets, performance scores, reaction-time information, game-based interaction, and associated attentional demands, the present design cannot determine the independent contribution of any single component. The observed between-group differences should therefore be interpreted as reflecting the combined gamified augmented-feedback intervention and its associated interactive features.

The greater improvement in dynamic balance observed with GBT is consistent with motor-learning theory, whereby interactive augmented feedback provides additional information about movement error beyond that available through intrinsic sensory feedback alone [17,26,27]. Real-time visual feedback may facilitate more rapid recognition and correction of postural errors, thereby reinforcing repeated sensorimotor adjustments across training [28]. Previous work has similarly demonstrated that feedback-guided and gamified balance interventions can improve postural control by increasing movement precision and the attentional demands associated with maintaining stability [29,30]. Importantly, improvements in the present study were demonstrated using the mSEBT rather than performance within the gamified training environment itself, suggesting that the adaptations transferred beyond the specific tasks used during training. Conventional wobble-board training also improved mSEBT performance relative to CON, confirming that the balance-training stimulus itself was effective. The additional improvement observed with GBT therefore suggests that gamified augmented feedback amplified the adaptations elicited by the balance-training stimulus.

The substantially greater increase in nocturnal RMSSD observed in GBT represents a particularly novel finding. RMSSD is a well-established marker of vagally mediated cardiac modulation, and the greater pre-to-post increase in GBT is consistent with favorable adaptation in vagally mediated cardiac regulation [20,21]. The mechanisms underlying this response cannot be determined from the present study, but several pathways are plausible. Balance training engages vestibular, cortical, and sensorimotor networks [31,32,33,34] that interact with central autonomic regulatory pathways [35,36,37]. However, because GBT and CBT received the same unstable-surface training stimulus, a purely balance- or vestibular-mediated explanation would predict a more comparable RMSSD response between the two conditions, which was not observed. The additional cognitive, attentional, and error-processing demands unique to GBT therefore represent a plausible contributor to the between-group difference, potentially through repeated engagement of cortical-autonomic networks involved in both cognitive control and vagal regulation [38,39,40]. These mechanistic interpretations remain hypothesis-generating and warrant direct investigation.

Gamification itself is unlikely to completely explain the superior adaptations observed with GBT. Rather, the interactive environment provided a mechanism for continuously increasing cognitive engagement, attentional demand, movement precision, and error correction [41,42]. Participants were required to respond to real-time visual information while simultaneously modifying postural behavior to achieve changing task objectives. By providing an on-screen target that indexed board orientation, the gamified system directed attention toward an external movement effect rather than toward the body, a manipulation associated with improved postural performance and retention under Wulf’s constrained action hypothesis [43,44]. Thus, gamification may be most appropriately viewed as a delivery mechanism through which augmented feedback and progressively challenging sensorimotor demands were integrated into balance training.

Training adherence was similarly high in both intervention groups (97.8%), indicating that the superior adaptations observed with GBT were not attributable to greater exposure to the prescribed training sessions. Although enjoyment and motivation were not directly assessed, the ability to achieve greater postural and vagally mediated cardiac modulation without increasing session duration, frequency, or overall training volume highlights the potential efficiency of augmenting an established exercise stimulus through real-time feedback rather than simply increasing training dose. Future studies should determine whether the effects of real-time augmented feedback on postural performance persist over longer training periods and whether perceived challenge, engagement, or adherence contribute to these adaptations.

Previous investigations of wobble-board training and augmented feedback have produced mixed findings, likely reflecting substantial differences in the type and intensity of feedback provided. Studies using relatively simple concurrent cognitive tasks have not consistently demonstrated additional balance benefits beyond conventional training [45], whereas interactive or gamified interventions providing real-time performance feedback have reported improvements in static and dynamic postural control across both healthy and clinical populations [46,47]. A methodological strength of the present design is that the physical training stimulus was matched as closely as practicable between GBT and CBT, permitting comparison of the combined gamified augmented-feedback intervention with conventional balance training without differences in exercise volume or modality. Exergaming interventions have also been associated with favorable HRV responses in older adults [48], although direct comparisons with the present population and training paradigm are limited. The current findings therefore extend this literature by demonstrating that real-time gamified augmented feedback can amplify dynamic balance improvement while producing a distinct nocturnal RMSSD response beyond that observed with conventional wobble-board training. Collectively, the divergent response patterns suggest that conventional wobble-board training was sufficient to improve dynamic balance, whereas the broader autonomic response emerged only when the same physical stimulus was paired with gamified augmented sensorimotor feedback.

The present findings have practical relevance because the gamified augmented-feedback intervention and its associated interactive features produced greater adaptations without increasing training frequency, duration, or overall training volume. Interactive feedback may therefore represent an efficient strategy for increasing the sensorimotor challenge of conventional balance training. Important strengths of the present investigation include the randomized controlled design, use of an active comparator performing an otherwise matched physical training protocol, assessor blinding, high intervention adherence, and objective assessment of both postural performance and nocturnal RMSSD. Several limitations should nevertheless be considered. The sample size was small (*n* = 10 per group), and the very large between-group effect estimates should be interpreted cautiously and confirmed in larger randomized trials. Participants were young, recreationally active adults, limiting generalizability to older adults, clinical populations, and highly trained athletes, and the study was not powered to examine sex-specific responses. The six-week intervention also precludes conclusions regarding long-term retention or translation to sport-specific performance, injury reduction, or broader functional outcomes. Nocturnal RMSSD can be influenced by sleep, recovery status, illness, alcohol or caffeine intake, and nonstudy physical activity. These day-to-day behaviors were not prospectively standardized beyond the overnight ring-wear instructions and therefore represent potential sources of residual variability. The study also did not include neuroimaging, electromyographic, kinetic, or kinematic measurements capable of directly evaluating the proposed mechanisms, and motivation, enjoyment, and perceived cognitive demand were not measured. Future studies should determine whether these adaptations persist following training cessation, translate to clinically meaningful outcomes, and are accompanied by measurable changes in the neural, neuromuscular, or autonomic regulatory pathways.

## 5. Conclusions

Six weeks of gamified balance training incorporating an interactive augmented-feedback environment produced greater improvements in dynamic postural control than conventional balance training or a non-training control condition in recreationally active adults. Conventional wobble-board training also improved dynamic balance relative to control, but the combined gamified augmented-feedback intervention and its associated interactive features substantially amplified this adaptation. Notably, nocturnal RMSSD increased to a greater extent following gamified training than following either conventional training or control, suggesting that the benefits of this combined intervention may extend beyond postural performance to favorable vagally mediated cardiac modulation. Because the interactive features were delivered as a bundled intervention, the present study cannot determine the independent contribution of any single component. Further research is warranted to determine the mechanisms underlying the observed RMSSD response and whether these adaptations persist over time or translate to clinical, functional, or injury-related outcomes.

## Figures and Tables

**Table 1:** Six-Week Balance Training Progression.

Week	Sessions	Primary Training Focus	Progression Strategy
1	1–3	Familiarization	Bilateral stance emphasizing postural control and board familiarization
2	4–6	Static Balance	Narrower base of support with increased postural precision
3	7–9	Controlled Weight Shifting	Multidirectional center-of-mass control in the anterior–posterior and medial–lateral planes
4	10–12	Dynamic Balance	Increased movement amplitude and multidirectional postural control tasks
5	13–15	Advanced Sensorimotor Control	Greater movement precision, faster target acquisition, and increased postural demands
6	16–18	Integrated Balance Performance	Highest level of balance complexity requiring rapid, accurate postural adjustments

**Table 2:** Baseline Characteristics by Randomized Group.

Characteristic	GBT (*n* = 10)	CBT (*n* = 10)	CON (*n* = 10)
Age (years)	20.00 ± 1.49	20.70 ± 0.95	20.70 ± 1.34
Height (cm)	171.00 ± 9.15	170.69 ± 6.31	171.02 ± 9.46
Body mass (kg)	73.72 ± 6.38	75.44 ± 6.67	73.80 ± 9.33
Nocturnal RMSSD (ms)	51.14 ± 4.09	51.59 ± 6.29	50.97 ± 6.17
mSEBT composite score (%)	87.89 ± 1.82	87.44 ± 4.89	88.57 ± 4.04

Values are mean ± SD. GBT = gamified balance training; CBT = conventional balance training; CON = control; RMSSD = root mean square of successive differences; mSEBT = modified Star Excursion Balance Test.

**Table 3:** Pre-to-Post Changes in Nocturnal RMSSD and Dynamic Balance.

**Panel A. Within-Group Pre-to-Post Responses.**													
**Outcome**		**Group**	**Pre** **Mean ± SD**		**Post** **Mean ± SD**		**Δ Change** **Mean ± SD**		**95% CI of Δ**		**% Change**		**Cohen’s *dz***
Nocturnal RMSSD (ms)		GBT	51.14 ± 4.09		58.06 ± 3.94		6.93 ± 1.86		5.60 to 8.25		13.71		3.73
		CBT	51.59 ± 6.29		52.60 ± 6.32		1.01 ± 1.38		0.02 to 2.00		2.00		0.73
		CON	50.97 ± 6.17		51.77 ± 7.15		0.80 ± 1.61		−0.35 to 1.95		1.44		0.50
mSEBT composite score (%)		GBT	87.89 ± 1.82		95.43 ± 1.55		7.54 ± 1.62		6.38 to 8.70		8.60		4.66
		CBT	87.44 ± 4.89		91.09 ± 4.77		3.65 ± 1.50		2.57 to 4.72		4.20		2.43
		CON	88.57 ± 4.04		89.03 ± 3.61		0.47 ± 1.46		−0.58 to 1.51		0.56		0.32
**Panel B. Holm-adjusted between-group comparisons of change scores.**													
**Outcome**	**Comparison**			**Mean Difference**		**95% CI**		**Adjusted *p***		**Hedges’ g**		**95% CI for g**	
Nocturnal RMSSD (ms)	GBT vs. CBT			5.92		4.37 to 7.46		<0.001		3.46		2.03 to 4.89	
	GBT vs. CON			6.12		4.49 to 7.76		<0.001		3.38		1.97 to 4.79	
	CBT vs. CON			0.21		−1.20 to 1.62		0.761		0.13		−0.75 to 1.01	
mSEBT composite score (%)	GBT vs. CBT			3.89		2.42 to 5.36		<0.001		2.39		1.21 to 3.56	
	GBT vs. CON			7.07		5.63 to 8.52		<0.001		4.40		2.72 to 6.09	
	CBT vs. CON			3.18		1.79 to 4.57		<0.001		2.06		0.95 to 3.17	

Nocturnal RMSSD: group × time interaction, Wald χ^2^(2) = 101.53, *p* < 0.001. mSEBT composite score: group × time interaction, Wald χ^2^(2) = 119.62, *p* < 0.001. Note. Values are mean ± SD. RMSSD = root mean square of successive differences; mSEBT = modified Star Excursion Balance Test; GBT = gamified balance training; CBT = conventional balance training; CON = control. Within-group standardized effects are Cohen’s dz. Between-group standardized effects compare change scores and are reported as Hedges’ g with 95% CIs. Pairwise *p*-values were adjusted using the Holm procedure.

## Data Availability

The datasets used and/or analyzed during the current study are available from the corresponding author upon reasonable request.

## Abbreviations

The following abbreviations are used in this manuscript:

HRV	Heart Rate Variability
RMSSD	Root Mean Square of Successive Differences between normal R-R intervals
GBT	Gamified Balance Training
CBT	Conventional Balance Training
CON	Control
mSEBT	modified Star Excursion Balance Test
PPG	Photoplethysmography
SD	Standard Deviation
CI	Confidence Interval
